# Harnessing entomopathogenic nematodes for sustainable pest management: mechanisms, challenges, and innovations

**DOI:** 10.3389/fpls.2026.1755114

**Published:** 2026-01-29

**Authors:** Amandeep Kaur, David Kihoro Sirengo, Pratibha Karki, Thomas O. Powers, Amanda M. V. Brown

**Affiliations:** 1 Department of Biological Sciences, Texas Tech University, Lubbock, TX, United States; 2 Department of Plant Pathology, University of Nebraska-Lincoln, Lincoln, NE, United States

**Keywords:** biological control, EPN, formulation, heterorhabditis, steinernema, stress tolerance mechanisms, sustainable agriculture, symbiotic bacteria

## Abstract

Entomopathogenic nematodes (EPNs) of the genera *Heterorhabditis* and *Steinernema* are increasingly recognized as potent biological control agents due to their ability to infect and kill diverse insect pest taxa through a symbiotic partnership with insect-pathogenic bacteria. Over the last decades, substantial progress has been made in improving EPN field performance through advances in formulation and application methods, use of biodegradable polymers and nanocarriers, and elucidation of stress tolerance mechanisms. However, despite their proven efficacy, large-scale commercialization of EPNs remains limited by high production costs, formulation instability, and environmental constraints. While numerous reviews have separately addressed EPN biology, mass production, or field application independently, a critical and integrative synthesis linking molecular mechanisms, and formulation strategies remains lacking. This review synthesizes current understanding of EPN biology with emphasis on molecular mechanisms governing host localization, invasion, and immune suppression, as well as their biotic ecological interactions within soil environments. We also discuss advances in stress tolerance mechanisms, innovations in formulation, and outline future research priorities needed to develop ecologically resilient EPN-based biocontrol products. As agriculture shifts toward more regenerative and environmentally sustainable systems, a comprehensive understanding of EPN biology, full ecological potential of EPN-bacteria partnerships holds promise not only for effective pest suppression but also for advancing fundamental understanding of host-microbe interactions and ecosystem resilience.

## 1 Introduction

The global population is projected to increase significantly by 2050 (United Nations, 2017), warranting a substantial increase in crop productivity through sustainable intensification and the adoption of innovative pest management strategies to ensure global food security. In this context, as agriculture shifts toward ecologically informed approaches, biological control has emerged as a cornerstone of environmentally sound, sustainable pest management. This paradigm shift has paved the way for the exploitation of naturally occurring beneficial microorganisms as alternatives and complements to synthetic chemical pesticides. As a result, the adoption of entomopathogenic nematodes (EPNs) as biocontrol agents against major agricultural insect pests is increasing across diverse cropping systems worldwide (Modic et al., 2020; Fallet et al., 2024; Polat et al., 2024; Ramakuwela et al., 2025). The successes of EPNs as biological control agents have been well demonstrated in various cropping systems against different insect taxa. In particular, EPNs have been effective in controlling stem flea beetle (*Psylliodes chrysocephala*) in winter oilseed rape (Godina et al., 2023), Pumpkin fruit fly (*Bactrocera tau*) in Chinese cucumber (Liu et al., 2025), false codling moth (*Thaumatotibia leucotreta*) in citrus (Moore et al., 2024), and codling moth (*Cydia pomonella*) in apple (Odendaal et al., 2016) among others. Moreover, compatibility of EPN with synthetic insecticides has been evaluated primarily through invitro bioassays, revealing species- and formulation-specific responses (Krishnayyaand and Grewal, 2002; Srivastava et al., 2011; Sabino PH de et al., 2014; Mahmoud et al., 2016; Wu et al., 2017; Dias S da et al., 2024). However, chemical compatibility with formulation components and application strategies needs to be tested under realistic agroecosystem conditions.

Beyond their practical applications, EPNs also serve as model organisms for advancing fundamental research on microbial mutualism and ecological interactions (Campos-Herrera et al., 2012; Tarasco et al., 2023; Stock et al., 2025). Notably, among EPN taxa, the infective juveniles (IJs) of *Heterorhabditis* and *Steinernema*, which maintain obligate symbioses with gammaproteobacteria of the genera *Photorhabdus* and *Xenorhabdus*, respectively, are the most intensively studied due to their effectiveness in biocontrol and relevance in both research and commercial applications. Within this highly specialized partnership, the nematode provides physical protection for its bacterial symbiont against environmental stresses such as temperature changes, moisture loss, and harmful microbes, and then releases the symbiont into the insect hemocoel upon infection (Stilwell et al., 2018). Once inside the host, these symbionts produce secondary metabolites and various toxins (Eleftherianos et al., 2007; Hinchliffe et al., 2010; Tobias et al., 2017) that suppress immune defenses and kill the host through septicemia, thereby creating a conducive environment for the nematode’s feeding and reproduction in the cadaver, facilitating later reacquisition of the bacteria before nematodes emerge as new IJs (Tarasco et al., 2023; Glazer et al., 2025). The insecticidal, antimicrobial and immunomodulatory roles of these secondary metabolites have been comprehensively reviewed elsewhere (Bode, 2009; Stock et al., 2017; Liao et al., 2025). Their application, however, is limited by host dependence and unresolved toxicity concerns, underscoring the need for systematic evaluation of stability, safety, and delivery strategies. Traditionally, this partnership has been viewed as species-specific, with each nematode species maintaining a single coevolved symbiont. Experimental evidence shows that these partnerships are tightly governed by partner recognition and fidelity and that pairing nematodes with non-cognates reduces fitness, virulence, and bacterial carriage (McMullen et al., 2017). Nonetheless, recent studies revealed the coexistence of other bacterial species with the primary symbiont. These secondary or facultative associates may enhance infective juvenile (IJ) insecticidal activity and expand the nematode’s host range (Pervez et al., 2020; Maher et al., 2021; Sugiyama and Hasegawa, 2025).

In addition to causing insect mortality, application of EPN in soil has been shown to modulate plant antioxidant defenses by influencing the activities of key enzymes such as peroxidases and catalase (Jagdale et al., 2009). For instance, the chemical cues such as ascarosides and 2,3-butanediol released by EPN-symbiont complex trigger oxidative bursts in root tissues, leading to activation of salicylic acid and jasmonic acid signaling pathways (Jagdale et al., 2009; Manohar et al., 2020). These hormonal cascades stimulate the expression of defense-related genes like *PR-1* (Helms et al., 2019; Wang et al., 2025) and enhance enzymatic scavenging of reactive oxygen species through increased peroxidase and catalase activity. These multitrophic interactions reflect the ecological versatility of EPNs and their growing importance in integrated pest management (IPM) frameworks. Their effectiveness can be further augmented through co-application with other biocontrol agents, such as entomopathogenic fungi, where synergistic interactions frequently result in enhanced pest suppression and increased crop productivity (Sáenz-Aponte et al., 2020; Půža and Tarasco, 2023). The remarkable successes of EPNs across diverse cropping systems demonstrate their potential as reliable biological control agents and alternatives to chemical pesticides. However, realizing their full potential requires overcoming challenges related to formulation, shelf life, field persistence, regulatory policies, and large-scale delivery systems (Blanco-Pérez et al., 2025). Several comprehensive reviews have examined EPNs with emphasis on EPN biology, mass production, application, field efficacy and challenges (Shapiro-Ilan and Dolinski, 2015; Koppenhöfer et al., 2020; Abd-Elgawad, 2023; Tarasco et al., 2023). However, most existing reviews treat EPN biology, symbiosis, formulation strategies as largely independent topics. This review aims to bridge this gap by evaluating how molecular mechanisms underlying host immune suppression, stress tolerance affects field performance and commercialization.

## 2 Molecular mechanisms of EPNs on insect infection

### 2.1 Host localization, foraging strategies, and invasion

**Figure 1:**
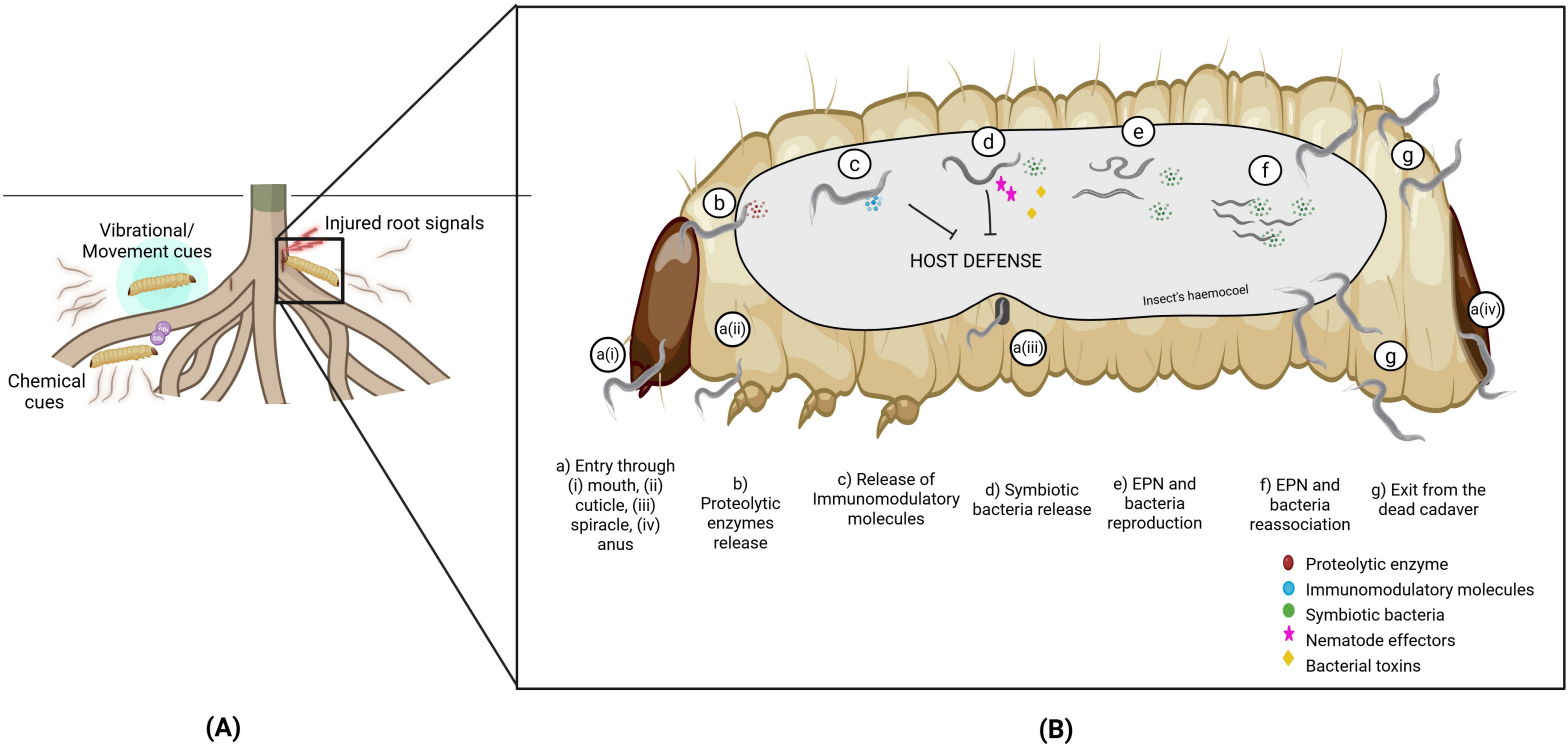
Schematic representation of **(A)** host-finding and **(B)** infection by entomopathogenic nematodes. Figure was created using BioRender.

Successful infection by EPNs relies on host localization mediated by chemical, physical, and other environmental cues (**Figure 1A**) (O’Halloran and Burnell, 2003; Ali et al., 2010; Hallem et al., 2011; Filgueiras et al., 2016; Laznik and Trdan, 2016; Baiocchi et al., 2017; Laznik et al., 2020; Zhang et al., 2021). Among the important chemical cues are the paired BAG sensory neurons that detect olfactory signals such as CO_2_, a byproduct of insect respiration. In *Heterorhabditis bacteriophora*, BAG neuron ablation abolishes chemotaxis toward the greater wax moth, *Galleria mellonella*, confirming the essential role of BAGs in host localization (Hallem et al., 2011). BAG neurons also contribute to nictation-triggering circuits in ambusher EPN species like *Steinernema carpocapsae* (Hallem et al., 2011). In addition to insect-derived signals, EPNs also respond to herbivore-induced plant volatiles (HIPVs) through chemosensory neurons (Rasmann et al., 2005; Degenhardt et al., 2009). Root-derived compounds such as carvone have been shown to activate *S. carpocapsae*, suggesting that plant volatiles can trigger specific chemosensory responses in these nematodes (Bai et al., 2024). Although the underlying neuronal circuits in EPNs remain poorly characterized, evidence from the model nematode, *Caenorhabditis elegans*, suggests that attraction and repulsion behaviors are mediated by complex interneuronal networks (Hart and Chao, 2010; Guillermin et al., 2017).

While these sensory mechanisms drive host detection, the specific cues used for host localization can vary depending on the EPN’s foraging strategy. Cruiser EPN species (e.g., *H. bacteriophora*) track long-range chemical, vibrational, and electrical signals, while ambushers (e.g., *S. carpocapsae*) use nictation to intercept mobile hosts (Lortkipanidze et al., 2016; Grunseich et al., 2021). Electrical fields generated by insect movement or root injury can elicit species-specific directional responses (Rasmann et al., 2005; Grunseich et al., 2021). For instance, the cruiser *Steinernema glaseri* moves toward higher electrical potentials, while the ambusher *S. carpocapsae* is attracted to lower potentials (Shapiro-Ilan et al., 2009; Shapiro-Ilan et al., 2012). Seismic cues produced by insect movement help detect hosts, particularly when chemical gradients are disrupted in organic-rich soils (Torr et al., 2004).

Following localization, host invasion is a critical and often challenging step in the infection process. EPNs utilize multiple routes to enter their hosts, and penetration routes vary among insect species. The choice of penetration pathway is both species- and host-specific (Batalla-Carrera et al., 2014; Kallali et al., 2024). Typically, EPNs invade through natural openings such as the mouth, spiracles, or anus, but some species can also directly penetrate the insect cuticle (Dowds and Peters, 2002).

### 2.2 Overcoming the insect immune system

While penetration marks the beginning of an infection process, the survival of EPNs within their hosts depends on their ability to bypass host immunity through coordinated interactions with their symbiotic bacteria (**Figure 1B**) (Ciche and Ensign, 2003; ffrench-Constant et al., 2007). Upon invasion, IJs of *H. bacteriophora* use their anterior tooth to pierce host tissues while secreting proteolytic enzymes that degrade structural barriers. On the other hand, *Steinernema* species degrade midgut epithelia with enzymes before they can enter the hemocoel (Ciche and Ensign, 2003; Balasubramanian et al., 2010). Immediately after penetration into the hemocoel, the IJs shed their second-stage cuticle, exposing a dynamic surface coat that masks immunogenic epitopes and releases small immunomodulatory molecules (Wang and Gaugler, 1999), an early immune evasion strategy that suppresses host recognition and prepares the hemocoel for bacterial release and establishment.

Once in the hemocoel, IJs release effector molecules via their excretory-secretory glands, which target both humoral and cellular immunity. Among these effectors, some of the most important examples include proteolytic enzymes that suppress the prophenoloxidase (pro-PO) cascade, therefore blocking melanization and nodulation. Similarly, a Kunitz-type protease inhibitor (Sc-KU-4) disrupts hemocyte encapsulation and aggregation (Balasubramanian et al., 2010; Chang et al., 2019). As a result, transient immune paralysis is induced, halting the host defense response and enabling the establishment of IJs. This process creates favorable conditions for the subsequent release and proliferation of the EPN’s symbiotic bacteria within the host (Lu et al., 2017). Once released from the nematode gut, the symbionts reinforce and extend host suppression through multiple molecular pathways. For instance, the symbiotic bacterium *Xenorhabdus nematophila* inhibits Toll and IMD (immune deficiency) signaling by suppressing eicosanoid biosynthesis, thereby halting the production of antimicrobial peptides (AMPs) such as cecropins and lysozymes (Park and Kim, 2000; Hwang et al., 2013).

Moreover, extracellular proteases from *Xenorhabdus* degrade pre-existing AMPs, hence enhancing humoral suppression (ffrench-Constant et al., 2007; Balasubramanian et al., 2010). Similarly, the symbionts *Photorhabdus luminescens* and *X*. *nematophila* secrete rhabduscin. This phenoloxidase inhibitor suppresses melanization and Mcf-family toxins and induces death of hemocytes, the primary immune cells in the hemolymph of invertebrates responsible for defense against pathogens, foreign entities, and gut epithelial cells (Dowling et al., 2004; Crawford et al., 2012). Serralysin-type proteases such as PrtA further degrade immune-related proteins, thereby enhancing systemic infection (Felföldi et al., 2009). These events later lead to a collapse of humoral and cellular defenses, resulting in rapid septicemia in the target host (Forst et al., 1997; da Silva et al., 2020).

Interestingly, several studies have demonstrated the pathogenicity of axenic EPNs under laboratory conditions. For instance, Ehlers et al. (1997) investigated the pathogenicity of the *Steinernema feltiae*-*Xenorhabdus bovienii* complex to the marsh crane fly, *Tipula oleracea*, and *G*. *mellonella* larvae by injecting dauer IJs using monoxenic nematodes (with bacteria), axenic nematodes (bacteria-free), and the symbiotic bacteria alone. Axenic IJs were less pathogenic than monoxenic IJs in both insects, indicating that bacterial symbionts significantly enhance virulence. Similarly, when axenic cultures of *G*. *mellonella* larvae were treated with axenic IJs of *H*. *bacteriophora* and *S*. *carpocapsae*, all larvae were killed by *S*. *carpocapsae*, whereas *H*. *bacteriophora* failed to cause mortality despite a few nematodes being recovered from the hemocoel (Han and Ehlers, 2000). Although these results represent partial pathogenic success, the nematode-bacterium association remains essential for complete pathogenicity and successful reproduction (Bisch et al., 2015). These results support the potential of EPNs as candidate model systems for studying the molecular mechanisms of parasitism and insect immune defense.

## 3 Stress tolerance mechanisms and environmental persistence challenges

Despite advances in the biology, development, and deployment of EPNs, their integration into IPM programs is constrained by interrelated challenges ranging from formulation and application to environmental limitations. The following sections highlight the stress tolerance and environmental persistence mechanisms, and the challenges of formulation and application constraints that affect EPN-based biocontrol.

### 3.1 Stress tolerance and environmental persistence

EPNs encounter both abiotic and biotic stresses in the environment. Among these, extreme temperatures, desiccation, and UV radiation represent key abiotic challenges, while microbial antagonists constitute major biotic threats. The field efficacy of EPNs as biological control agents largely depends on their ability to withstand diverse biotic and abiotic stresses. The environmental persistence is further determined by stress tolerance mechanisms that may be species- or strain-specific as well as by interactions with soil type and the availability of suitable hosts. IJs acclimate to thermal stress by accumulating trehalose (Grewal and Jagdale, 2002; Jagdale and Grewal, 2003; Grewal et al., 2006) and by reducing metabolic activity (Brown and Gaugler, 1996; Andalo et al., 2011; Ali and Wharton, 2013). Although IJs possess mechanisms to lower their lipid reserves, species-specific differences exist in how metabolic adjustments occur. For instance, *Heterorhabditis megidis* reduces production of proteins involved in metabolism and protein synthesis, whereas *H. carpocapsae* downregulates proteins involved in intermediary metabolism and oxidative phosphorylation (Jagdale and Grewal, 2003; Andalo et al., 2011).

Under severe dehydration, IJs enter a state of anhydrobiosis, and osmoregulant molecules such as, heat-stable, water-stress-related protein (DESC47), and heat-shock protein (HSP40) are synthesized (Solomon et al., 2000; Gal et al., 2003; Somvanshi et al., 2008). In *S. feltiae*, desiccation induced a two-fold increase in trehalose content (Solomon et al., 1999). Furthermore, the induction of casein kinase (CK2) has been observed, which is hypothesized to participate in the transcriptional activation of nucleosome-assembly protein (NAP-1) (Gal et al., 2003). While UV tolerance mechanism in EPNs is unclear, the general assumption is that UV tolerance mechanisms parallel those described in *C. elegans*. In *C. elegans*, UV light is detected and avoided through four sensory neurons (ASJ, ASK, AWB, and ASH) with the neurotransmitter glutamate receptors (*glc-3, mgl-1, mgl-2*) expressed in ASH and ASK. These neurons play key roles in mediating this response (Bargmann, 2006; Ozawa et al., 2022).

In addition to species- and strain-specific physiological stress responses, ecological adaptation plays a critical role in determining EPN persistence and field efficacy. Locally adapted beneficial microbes exhibit superior tolerance to changing weather and climatic conditions, enhanced dispersal, and form a more stable association with the native soil microbiota (Campos-Herrera et al., 2013). Native EPNs have been shown to exhibit strong virulence against pests for major crops and turf grasses (Yadav and Lalramliana, 2012; Sun et al., 2021; Thakur et al., 2022; Tumialis et al., 2023; Fallet et al., 2024; Koppenhöfer and Luiza Sousa, 2024) and show greater potential when integrated with chemical control strategies (Shields et al., 2018; Tomar and Thakur, 2022). In addition, the EPN-symbiont complex produces a myriad of natural compounds that include scavenger deterrent factors (SDFs) with antifungal, insecticidal, and antibacterial properties. These metabolites not only deter scavengers from colonizing insect cadavers but also help the EPNs and their symbiotic bacteria overcome host immune defenses, ensuring successful infection and reproduction (Furgani et al., 2008; Gulcu et al., 2012; Fang et al., 2014; Hazir et al., 2016; Tobias et al., 2017; Gulcu et al., 2018).

### 3.2 Formulation and application

Successful commercialization of EPNs depends on their formulation and handling. However, one major obstacle limiting their field performance arises during formulation (Chen and Glazer, 2005). To increase EPN shelf life and efficacy, EPN treatments have been formulated using several carriers such as liquid concentrates, alginate gels, water-dispersible granules, synthetic sponge, and vermiculite applied in aqueous suspension through agricultural sprayers or irrigation systems (Georgis, 1990; Grewal, 2000) (**Table 1**). Traditionally, early work on water-dispersible granules showed enhanced improvements in EPN longevity and ease of handling compared with aqueous formulations (Grewal, 2000). Nonetheless, most aqueous products require refrigeration to maintain IJ viability, which results in increased production and transport costs and limits use in regions without reliable cold storage (Chen and Glazer, 2005; Karimi et al., 2025).

**Table 1:** Entomopathogenic nematode (*Steinernema* and *Heterorhabditis* spp.) formulation types, mechanisms governing efficacy and limitations, and directions for future research.

Formulation type	Adjuvant/additive	EPN species formulated	Mechanism of success (physiological/physical)	Mechanism of failure/limitation	Future optimization priorities	References
Activated charcoal gels	Activated carbon powder, wetting agents, buffers	*Steinernema feltiae*	Porous carbon matrix suspended nematodes, reduced sedimentation and helped adsorb toxic metabolites in storage.	Limited shelf-life and low instability at room temperature.	Integrate humectants and antioxidants, optimize carbon:nematode ratios	(Yukawa and Pitt, 1985; Grewal, 1998)
Synthetic sponge	Polyether-polyurethane sponge sheets or cubes	*Heterorhabditis bacteriophora, H. indica*	The porous sponge matrix provides a large surface area that promotes oxygen exchange and maintains moisture, mimicking the nematode’s natural soil habitat. Sponge perforations limit sedimentation and support IJ survival during storage.	Recovery of nematodes from the sponge matrix is difficult, and formulation density is constrained by sponge pore capacity, thereby limiting scalability and uniform field application.	Develop biodegradable or dissolvable sponge matrices and optimize their pore size to improve IJ loading and release	(Strauch et al., 2000)
Wheat gluten granules (‘Pesta’)	Wheat flour, bentonite, kaolin, peat, 0.2% formaldehyde.	*S*. *carpocapsae*	Wheat flour/gluten matrix slowly removed free water and induced quiescence and preserved lipid reserves within granules.	Low survival rate after 6 weeks of storage at 21 °C. Moreover, high humidity and flour matrix favored fungal and or bacterial growth.	Explore alternative cereal matrices less supportive of saprophytes, improve moisture control, and add antifungal and antibiotic agents.	(Connick et al., 1993; Connick et al., 1994)
Calcium alginate beads	Glycerol (18%), CaCO_3_·2H_2_O, CaCl_2_ (0.3-3% w/v), gluconolactate, xanthan gum.	*H. bacteriophora*, *H*. *heliothidis*, *S*. *carpocapsae*, *S*. *feltiae*	solid Ca^2+^ alginate beads supplemented with glycerol retain IJs during storage and ensure a steady release when applied in soil. Glycerol-induced metabolic arrest in IJs result in quiescence and total retention of IJs when added to beads made with 0.5% sodium alginate and 2% CaCl_2_·2H_2_O solutions.	Dense or oversized beads can limit oxygen diffusion and nematode escape, leading to hypoxia and low field release.	Optimize bead size and porosity, design beads for mechanized delivery like banding or in-furrow applications.	(Hussein and Abdel-Aty, 2012; Kim et al., 2015)
Water-dispersible granules	Diatomaceous earth, hydroxyethylcellulose, amorphous silica, fumed hydrophobic silica, lignosulfonate, starch, pregelatinised starch, pregelled attapulgite clay.	*S*. *carpocapsae*, *S*. *feltiae*, *S*. *scapterisci*, *S*. *riobravis*	Controlled desiccation in granules induces partial anhydrobiosis, therefore extending shelf-life at moderate temperatures. This formulation achieved 90% survival for 6 weeks at room temperature.	Rehydration can cause osmotic shock if too rapid and some species lose infectivity after long-term desiccation	Improve drying-rehydration protocols, include osmoprotectants, and improve granule dispersibility	(Silver et al., 1995; Grewal, 2000)
Polyacrylamide gels	Anhydrous polyacrylamide, vermiculite	*S*. *carpocapsae*	Hydrophilic polymer retains water and physically immobilizes IJs, preventing sedimentation and mechanical damage.	The gels are difficult to dissolve, and IJs have a short survival time at room temperature.	Develop degradable gels to enhance IJs release	(Bedding and Butler, 1994; Bedding et al., 2000; Leite et al., 2018)
Infected cadavers and coated infected cadavers	Kaolin-starch mixture, unflavored gelatin, inert clay, protective powders	*H*. *bacteriophora*, *H. baujardi S*. *carpocapsae*	Host cadaver protects IJs from desiccation and UV. The coatings further reduce mechanical damage and enhance handling.	Cadavers can rupture or mold during storage, and producing them at large scale is bulky and labor-intensive.	Automate cadaver coating and engineer delayed emergence storage methods	(Shapiro-Ilan et al., 2001; Shapiro et al., 2003; Ansari et al., 2009; Valle et al., 2009; Shapiro-Ilan et al., 2010b; Raja et al., 2015)
Protective gel-coating (Barricade^®^ fire gel)	Polyacrylate fire gel	*H*. *floridensis*, *S*. *carpocapsae, S. riobrave, H*. *bacteriophora*, *S*. *tophus*, *S*. *innovationi*	Highly hydrated gel layer protects IJs from UV and desiccation on foliage, which extends the survival window for host invasion.	High viscosity can increase pump pressure and shear stress in spray equipment.	Optimize gel concentrations for lower shear while maintaining protection of IJs. Redesign low-shear nozzle systems if needed.	(Shapiro-Ilan et al., 2010a; Shapiro-Ilan and Goolsby, 2021; Zulu et al., 2025)
Oil-in-water emulsions (vegetable oil)	Vegetable oils, emulsifiers, non-ionic surfactants, stabilizers	*H*. *bacteriophora*, *S*. *carpocapsae*, *S*. *websteri*	Oil droplets reduce cuticular water loss from IJs and can improve persistence on exposed surfaces.	Unstable emulsions or phase separation can concentrate nematodes in oil-rich phases and reduce oxygen availability.	Optimize oil:water ratio for oxygen and sprayability and stabilize emulsions across hard water and agitation regimes	(Aquino-Bolaños et al., 2019)
Chitosan additives	Chitosan matrices	*S*. *carpocapsae*	A chitosan matrix can enhance spray retention and may stimulate plant defenses, thereby indirectly supporting EPN persistence and performance.	The matrix’s solubility depends on pH. Moreover, poor dissolution or precipitation can cause clogging and uneven nematode distribution.	Standardize chitosan chemistry and buffer to a stable pH. Also, test blends with compatible polymers for better sprayability and release	(Llácer et al., 2009)
Carboxymethyl cellulose (CMC) gels	Sodium polyacrylate, carboxymethyl cellulose, vermiculite coating, inorganic salts (NaCl, KCl, MgSO_4_).	*S*. *carpocapsae*, *H*. *bacteriophora*	CMC gel holds moisture around IJs and can be applied as a targeted gel plug on plants (e.g. maize whorl). It also slows evaporation and sedimentation.	Too high CMC concentration reduces sprayability; gel can still dry out under extreme heat without additional protectants and hard water can affect performance.	Optimize viscosity with humectants and UV protectants for application methods and validate performance under realistic field relative humidity/temperature conditions	(Kary et al., 2021; Fallet et al., 2024; Karimi et al., 2025)
Titanium Pickering emulsion (TPE)	TiO_2_ nanoparticles, mineral/vegetable oil	*S*. *carpocapsae*	TiO_2_ nanoparticles in oil-in-water Pickering emulsions form a reflective coating that reduces UV damage and water loss while maintaining viability.	Nanoparticle aggregation or inappropriate oil:water ratios can reduce coating uniformity and harm IJs survival.	Optimize nanoparticle size and surface chemistry	(Kotliarevski et al., 2022; Ramakrishnan et al., 2023)
Silica-Pickering emulsion gel (SPEG)	Silica nanoparticles, oil droplets, gelled aqueous phase	*S*. *carpocapsae*	Silica-based Pickering gel slows desiccation and maintains IJs on foliage for extended periods under solar exposure.	Formulation cost and sensitivity to water chemistry can limit large-scale adoption at present.	Develop cheaper silica/clay hybrids and simplify mixing for end users	(Ramakrishnan et al., 2023)
Potassium polyacrylate hydrogel (PPH)	Potassium polyacrylate superabsorbent polymers, other optional fillers	*H*. *bacteriophora*, *S*. *tophus*, *S*. *innovationi*	Superabsorbent hydrogel releases water gradually in soil, helping IJs disperse from the application point.	High salinity or fertilizer ions can collapse the gel and reduce water-holding capacity.	Formulate for salt tolerance; blend with organic carriers to buffer ions	(Zulu et al., 2025)
Diatomaceous earth (DE) pellets	Diatomaceous earth (Celite 209), hygroscopic clays, binders.	*S*. *glaseri*	Porous DE matrix controls evaporation and can induce partial anhydrobiosis while providing mechanical protection.	Non-uniform pore size and low moisture can over-dry some IJs, reducing post-rehydration infectivity.	Standardize porosity and moisture of DE pellets; add moisture buffers like SAP; optimize rehydration procedures	(Matadamas-Ortiz et al., 2014; Cortés-Martínez et al., 2016)
Liquid-core alginate capsules	Alginate shell, Ca²^+^ crosslinker, gluconolactate, buffers, and optional viscosity modifiers.	*H*. *bacteriophora*, *S*. *carpocapsae*, *S*. *feltiae*	The liquid core keeps IJs active while the alginate shell protects them from desiccation and permits targeted root-zone release.	Capsules can leak or allow premature IJ escape during storage, and IJs have a limited shelf-life at room temperature.	Incorporate multilayer shells or coatings to minimize leakages	(Hiltpold et al., 2012; Kim et al., 2021)
Bait formulations (edible gels)	Alginate-based edible gels + phagostimulants	*S*. *feltiae*, *H*. *heliothidis*	Edible calcium-alginate gels incorporate phagostimulants to promote ingestion by the target insect larvae which are less exposed to surface penetration via gut route.	Gels can desiccate and lose attractiveness which reduces palatability; field performance is sensitive to bait placement and pest behavior.	Improve moisture retention by adding humectants; design pest-specific attractants to promote ingestion; integrate the baits with monitoring to time placement	(Navon et al., 1998; Navon et al., 2002)
Hygroscopic attapulgite clay ‘sandwich’ granules	Attapulgite clay layers	*H*. *heliothidis*, *S*. *bibionis*, *S*. *feltiae*, *S*. *glaseri*	Layered attapulgite clay and nematode slurry induced controlled desiccation and partial anhydrobiosis for storage with survival time of 8 weeks at 23 °C.	Short shelf-life, nozzle clogging in spray equipment, and low nematode-clay proportion led to the discontinuation of this commercial product.	Re-engineer the formulation as soil-applied granules rather than sprayable	(Bedding, 1988; Grewal, 2002)
Hydrogenated vegetable oil paste (microgels)	Hydrogenated oils, mono-/diglycerides	*S*. *carpocapsae*	A hydrophobic matrix slows water loss and buffers thermal fluctuations during shipment of IJs.	Low oxygen diffusion through the paste can limit IJ survival under suboptimal storage.	Introduce microchannels or porous fillers to improve oxygen circulation within the paste	(Chang and Gehret, 1992; Chang and Gehret, 1995)
Pre-infected live hosts (living insect bombs)	Live insect hosts as delivery cargo	*S. carpocapsae*	Live infected hosts can move and actively deliver IJs into cryptic habitats before nematode emergence and help in delivering inoculum directly to target zones.	Biologically complex multi-organism rearing makes it disadvantageous; timing when to apply and regulatory concerns limit practical use.	Develop predictive timing models to enhance efficacy; restrict application to niche pests like borers where benefits justify complexity	(Gumus et al., 2015)

EPN-infected cadavers have been explored as natural carriers (Shapiro and Glazer, 1996; Shapiro-Ilan et al., 2001). The IJs emerging from infected hosts disperse efficiently through soil, are highly infective, and provide better pest control than nematodes applied in suspension (Shapiro and Lewis, 1999; Perez et al., 2003; Shapiro-Ilan et al., 2003). For instance, field trials using *H. bacteriophora*-infected cadavers effectively reduced infestations of the sweet potato weevil, *Cylas formicarius*, and the guava weevil, *Conotrachelus psidii*. The reliability of cadavers as a dispersal agent, however, is limited because they desiccate and rupture during production and storage. These challenges were addressed by formulating cadavers with protective materials that prevent sticking and mechanical damage (Shapiro-Ilan et al., 2003). However, the influence of cadaver age on stability and efficacy remains unclear.

Recent advancements concentrate on improving stability in ambient conditions. For example, Darsouei et al. (2025) demonstrated that several EPN species encapsulated in calcium-alginate beads survived up to three months at room temperature under laboratory conditions. Similarly, hydrogel and emulsion capsules were shown to improve persistence *of S. feltiae* against the yellow mealworm beetle, *Tenebrio molitor*, under laboratory and field conditions (Perier et al., 2025). Other studies have demonstrated that encapsulation in alginate gels and hydrophilic colloids also shields IJs from desiccation and UV stress (Kim et al., 2021). Additionally, carboxymethyl cellulose (CMC), a water-soluble cellulose derivative, has emerged as an effective substrate for EPN formulation, particularly for *S. carpocapsae* (Kary et al., 2021). Despite these advances, cost-effective commercialization remains a challenge (San-Blas, 2013; Ramakuwela et al., 2016). Even with successful formulations, effectiveness ultimately depends on proper handling and field application.

During application, IJs are introduced to stressors such as tank sedimentation (Schroer et al., 2005), nozzle shear or pressure damage (Moreira et al., 2013), and post-application desiccation mortality (Platt et al., 2018). Several adjuvants and surfactants have been formulated recently to improve efficiency. The addition of xanthan gum or potassium alginate resulted in a two-fold increase of insect mortality at 80% relative humidity (RH) and a five-fold increase at 60% RH, while the mixtures of 0.3% xanthan or alginate with 0.3% surfactants further improved efficacy for foliar application of *S*. *carpocapsae* against diamondback moth larvae (Schroer et al., 2005). Similarly, *S. carpocapsae* formulated in an alkyl polyglycoside polymeric surfactant reduced the tomato pinworm, *Tuta absoluta*, larvae populations within two weeks post-treatment (Kajuga et al., 2025). Nonetheless, the efficacies of these adjuvants or surfactants are not consistently reliable. Some, for instance, increase longevity and enhance host invasion, whereas others reduce infectivity (Beck et al., 2013).

## 4 Future directions and opportunities

Given the growing demand for safer food and environmentally sustainable pest management practices, the commercial potential of EPNs is expanding (Dara, 2019). However, unlike chemical pesticides, their commercialization is constrained by multiple factors (Koppenhöfer et al., 2020). Addressing these limitations requires a stronger focus on biological and ecological factors that influence EPNs’ performance. Systematic screening of native EPN isolates, coupled with formulation optimization could further enhance persistence while reducing reliance on protective encapsulants.

Beyond strain selection, interactions among EPNs, their bacterial symbionts, and soil microbial communities may influence EPN efficacy. Nevertheless, the underlying mechanisms remain poorly understood. Therefore, metagenomic profiling of soil microbial community in soils with contrasting EPN efficacies can identify microbial taxa that promote EPN success or suppress their activity and enable the design of synthetic microbial communities that can be co-inoculated with EPNs to prime the soil environment, ensuring higher success rate in non-native soils. On the other hand, limited efficacy or genetic diversity in natural strains can be addressed through genomic-assisted breeding and molecular genetic approaches, including mutagenesis, transgenesis, and targeted gene modification. Over the past decades, these approaches have been applied to enhance EPN stress tolerance and longevity (Shapiro et al., 1997; Hashmi et al., 1998; Vellai et al., 1999; Sumaya et al., 2018). Selective breeding, however, can introduce fitness trade-offs, as demonstrated by altered reproduction, dispersal, and storage stability in modified *S. carpocapsae* strains (Gaugler et al., 1990; Bal et al., 2014). Future efforts to develop host-specific EPNs may extend their efficacy beyond soil-dwelling insects to targeted pests across various cropping systems (Lacey and Georgis, 2012). The application of precise and advanced genetic engineering technologies such as CRISPR/Cas9 offers a pathway to precisely enhance EPN environment resilience through targeted modification.

Recent advancements in IJ recovery through biodegradable polymers and nanocarriers are emerging as promising tools for enhancing the EPN performance and tolerance under various field stress conditions. For instance, alginate-starch hydrogel increased *S. feltiae* persistence and infectivity across different water regimes (Perier et al., 2025), while titanium-dioxide-based Pickering emulsions maintained *S. carpocapsae* viability for up to 120 days (Kotliarevski et al., 2022). Future research should prioritize precision-based delivery systems that synchronize nematode release with pest phenology and prevailing microclimatic conditions compared to conventional aqueous sprays. EPN application via drip irrigation systems resulted in more uniform nematode distribution, improved persistence, and significantly greater control of onion thrips compared with standard foliar sprays (Metwally et al., 2025). Emerging AI-driven automatic and controlled treatment systems could further optimize site-specific deployment via drones or automated irrigation, targeting pest “hotspots” and reducing costs and environmental impact. To maximize field efficacy, EPNs should be applied during early morning or late evening to reduce UV exposure, in pre-moistened soils to enhance IJ motility, and using methods that minimize mechanical shear. When combined with improved formulations and targeted genetic enhancements, such synchronized, precision-based delivery can elevate EPNs from supplemental inputs to reliable, site-specific components of modern IPM programs.

## 5 Concluding remarks

In summary, EPNs offer a promising avenue for sustainable pest management, providing a biologically robust alternative or complement to synthetic chemical control. Continued innovation in formulation, strain selection, and application technologies, supported by insights from genomics, nanotechnology, and microbial ecology, will be essential to enhance their persistence and reliability under field conditions. Omics-based discovery of genes and regulatory pathways in EPNs can guide more precise, targeted improvements in strain performance. However, potential trade-offs between enhanced traits should be considered. Future innovations should also focus on ecological interactions and development of strategies that integrate EPNs with beneficial microbes to enhance EPN persistence, infectivity, and strain stability in field deployment.

## References

[B1] Abd-Elgawad M. M. M. (2023). Optimizing entomopathogenic nematode genetics and applications for the integrated management of horticultural pests. Horticulturae 9, 865. doi: 10.3390/horticulturae9080865

[B2] Ali F. Wharton D. A. (2013). Cold tolerance abilities of two entomopathogenic nematodes, *Steinernema feltiae* and Heterorhabditis bacteriophora. Cryobiology 66, 24–29. doi: 10.1016/j.cryobiol.2012.10.004, PMID: 23142823

[B3] Ali J. G. Alborn H. T. Stelinski L. L. (2010). Subterranean herbivore-induced volatiles released by citrus roots upon feeding by *Diaprepes abbreviatus* recruit entomopathogenic nematodes. J. Chem. Ecol. 36, 361–368. doi: 10.1007/s10886-010-9773-7, PMID: 20309617

[B4] Andalo V. Moino A. Maximiniano C. Campos V. P. Mendonca L. A. (2011). Influence of temperature and duration of storage on the lipid reserves of entomopathogenic nematodes. Rev. Colomb. Entomol. 37, 203–209. doi: 10.25100/socolen.v37i2.9075

[B5] Ansari M. A. Hussain M. A. Moens M. (2009). Formulation and application of entomopathogenic nematode-infected cadavers for control of *Hoplia philanthus* in turfgrass. Pest Manag. Sci. 65, 367–374. doi: 10.1002/ps.1699, PMID: 19165730

[B6] Aquino-Bolaños T. Ruiz-Vega J. Hernández Y. D. O. Castañeda J. C. J. (2019). Survival of entomopathogenic nematodes in oil emulsions and control effectiveness on adult engorged ticks (Acari: ixodida). J. Nematol. 51, 1–10. doi: 10.21307/jofnem-2019-001, PMID: 31115202 PMC6929640

[B7] Bai P. H. Yu J. P. Hu R. R. Fu Q. W. Wu H. C. Li X. Y. . (2024). Behavioral and molecular response of the insect parasitic nematode *Steinernema carpocapsae* to plant volatiles. J. Invertebr. Pathol. 203, 108067. doi: 10.1016/j.jip.2024.108067, PMID: 38278342

[B8] Baiocchi T. Lee G. Choe D. H. Dillman A. R. (2017). Host seeking parasitic nematodes use specific odors to assess host resources. Sci. Rep. 7, 6270. doi: 10.1038/s41598-017-06620-2, PMID: 28740104 PMC5524962

[B9] Bal H. K. Michel A. P. Grewal P. S. (2014). Genetic selection of the ambush foraging entomopathogenic nematode, *Steinernema carpocapsae* for enhanced dispersal and its associated trade-offs. Evol. Ecol. 28, 923–939. doi: 10.1007/s10682-014-9706-y

[B10] Balasubramanian N. Toubarro D. Simões N. (2010). Biochemical study and *in vitro* insect immune suppression by a trypsin-like secreted protease from the nematode Steinernema carpocapsae. Parasit. Immunol. 32, 165–175. doi: 10.1111/j.1365-3024.2009.01172.x, PMID: 20398179

[B11] Bargmann C. I. (2006). “ Chemosensation in C. elegans,” in WormBook: The online review of *C. elegans* biology. The C. elegans Research Community. Pasadena (CA). 1–29. doi: 10.1895/wormbook.1.123.1, PMID: PMC4781564 18050433

[B12] Batalla-Carrera L. Morton A. Shapiro-Ilan D. Strand M. R. García-del-Pino F. (2014). Infectivity of *Steinernema carpocapsae* and S. feltiae to larvae and adults of the hazelnut weevil, *Curculio nucum*: Differential virulence and entry routes. J. Nematol. 46, 281–286., PMID: 25276002 PMC4176411

[B13] Beck B. Brusselman E. Nuyttens D. Moens M. Pollet S. Temmerman F. . (2013). Improving foliar applications of entomopathogenic nematodes by selecting adjuvants and spray nozzles. Biocontrol Sci. Technol. 23, 507–520. doi: 10.1080/09583157.2013.777692

[B14] Bedding R. A. (1988). Storage of insecticidal nematodes. World Patent No. WO 88/08668. Geneva, CH: World Intellectual Property Organization.

[B15] Bedding R. A. Butler K. L. (1994). Method for the storage of entomopathogenic nematodes. WIPO Patent No. WO 94/05150. Geneva, CH: World Intellectual Property Organization.

[B16] Bedding R. A. Clark S. D. Lacey M. J. Butler K. L. (2000). Method and apparatus for the storage of entomopathogenic nematodes. WIPO Patent No. WO 00/18887. Geneva, CH: World Intellectual Property Organization.

[B17] Bisch G. Pagès S. McMullen J. G. Stock S. P. Duvic B. Givaudan A. . (2015). *Xenorhabdus bovienii* CS03, the bacterial symbiont of the entomopathogenic nematode *Steinernema weiseri*, is a non-virulent strain against Lepidopteran insects. J. Invertebr. Pathol. 124, 15–22. doi: 10.1016/j.jip.2014.10.002, PMID: 25315609

[B18] Blanco-Pérez R. San-Blas E. Rivera M. J. Campos-Herrera R. (2025). Population ecology of entomopathogenic nematodes: Bridging past insights and future applications for sustainable agriculture. J. Invertebr. Pathol. 211, 108313. doi: 10.1016/j.jip.2025.108313, PMID: 40107567

[B19] Bode H. B. (2009). Entomopathogenic bacteria as a source of secondary metabolites. Curr. Opin. Chem. Biol. 13, 224–230. doi: 10.1016/j.cbpa.2009.02.037, PMID: 19345136

[B20] Brown I. M. Gaugler R. (1996). Cold tolerance of Steinernematid and Heterorhabditid nematodes. J. Therm. Biol. 21, 115–121. doi: 10.1016/0306-4565(95)00033-X

[B21] Campos-Herrera R. El-Borai F. E. Duncan L. W. (2012). Wide interguild relationships among entomopathogenic and free-living nematodes in soil as measured by real time qPCR. J. Invertebr. Pathol. 111, 126–135. doi: 10.1016/j.jip.2012.07.006, PMID: 22841945

[B22] Campos-Herrera R. Pathak E. El-Borai F. E. Schumann A. Abd-Elgawad M. M. M. Duncan L. W. (2013). New citriculture system suppresses native and augmented entomopathogenic nematodes. Biol. Control. 66, 183–194. doi: 10.1016/j.biocontrol.2013.05.009

[B23] Chang D. Z. Serra L. Lu D. Mortazavi A. Dillman A. R. (2019). A core set of venom proteins is released by entomopathogenic nematodes in the genus Steinernema. PloS Pathog. 15, e1007626. doi: 10.1371/journal.ppat.1007626, PMID: 31042778 PMC6513111

[B24] Chang F. N. Gehret M. J. (1992). Insecticide delivery system and attractant. U.S. Patent No. 5141744. Washington, DC: U.S. Patent and Trademark Office.

[B25] Chang F. N. Gehret M. J. (1995). Stabilized insect nematode compositions. U.S. Patent No. 5401506. Washington, DC: U.S. Patent and Trademark Office.

[B26] Chen S. Glazer I. (2005). A novel method for long-term storage of the entomopathogenic nematode *Steinernema feltiae* at room temperature. Biol. Control. 32, 104–110. doi: 10.1016/j.biocontrol.2004.08.006

[B27] Ciche T. A. Ensign J. C. (2003). For the insect pathogen *Photorhabdus luminescens*, which end of a nematode is out? Appl. Environ. Microbiol. 69, 1890–1897. doi: 10.1128/AEM.69.4.1890-1897.2003, PMID: 12676661 PMC154793

[B28] Connick W. J. Nickle W. R. Vinyard B. T. (1993). Pesta”: New granular formulations for Steinernema carpocapsae. J. Nematol 25, 198–203., PMID: 19279759 PMC2619378

[B29] Connick W. Nickle W. R. Williams K. Vinyard B. (1994). Granular formulations of *Steinernema carpocapsae* (strain all) (Nematoda: Rhabditida) with improved shelf life. J. Nematol. 26, 352–359., PMID: 19279903 PMC2619510

[B30] Cortés-Martínez C. I. Ruiz-Vega J. Matadamas-Ortiz P. T. Lewis E. E. Aquino-Bolaños T. Navarro-Antonio J. (2016). Effect of moisture evaporation from diatomaceous earth pellets on storage stability of Steinernema glaseri. Biocontrol Sci. Technol. 26, 305–319. doi: 10.1080/09583157.2015.1104650

[B31] Crawford J. M. Portmann C. Zhang X. Roeffaers M. B. J. Clardy J. (2012). Small molecule perimeter defense in entomopathogenic bacteria. Proc. Natl. Acad. Sci. 109, 10821–10826. doi: 10.1073/pnas.1201160109, PMID: 22711807 PMC3390839

[B32] Dara S. K. (2019). The new integrated pest management paradigm for the modern age. J. Integr. Pest Manag., 10(1). doi: 10.1093/jipm/pmz010

[B33] Darsouei R. Karimi J. Stelinski L. L. (2025). Properties of enhanced calcium-alginate beads as a formulation for disseminating the entomopathogenic nematodes *Heterorhabditis bacteriophora*, *Steinernema carpocapase*, and Steinernema feltiae. J. Nematol 57, 20250020. doi: 10.2478/jofnem-2025-0020, PMID: 40469270 PMC12136810

[B34] da Silva W. J. Pilz-Júnior H. L. Heermann R. da Silva O. S. (2020). The great potential of entomopathogenic bacteria *Xenorhabdus* and *Photorhabdus* for mosquito control: A review. Parasit. Vectors. 13, 376. doi: 10.1186/s13071-020-04236-6, PMID: 32727530 PMC7391577

[B35] Degenhardt J. Hiltpold I. Köllner T. G. Frey M. Gierl A. Gershenzon J. . (2009). Restoring a maize root signal that attracts insect-killing nematodes to control a major pest. Proc. Natl. Acad. Sci. 106, 13213–13218. doi: 10.1073/pnas.0906365106, PMID: 19666594 PMC2726344

[B36] Dias S da C. de Brida A. L. Jean-Baptiste M. C. Leite L. G. Ovruski S. M. Lee J. C. . (2024). Compatibility of entomopathogenic nematodes with chemical insecticides for the control of *Drosophila suzukii* (Diptera: Drosophilidae). Plants 13, 632. doi: 10.3390/plants13050632, PMID: 38475479 PMC10934839

[B37] Dowds B. C. Peters A. (2002). " Virulence mechanisms." in Entomopathogenic Nematology. Ed. R. Gaugler (CABI, Wallingford), 79–98. doi: 10.1079/9780851995670.0079

[B38] Dowling A. J. Daborn P. J. Waterfield N. R. Wang P. Streuli C. H. Ffrench-Constant R. H. (2004). The insecticidal toxin Makes caterpillars floppy (Mcf) promotes apoptosis in mammalian cells: An insecticidal toxin promoting apoptosis. Cell Microbiol. 6, 345–353. doi: 10.1046/j.1462-5822.2003.00357.x, PMID: 15009026

[B39] Ehlers R. U. Wulff A. Peters A. (1997). Pathogenicity of axenic *Steinernema feltiae*, *Xenorhabdus bovienii*, and the bacto–helminthic complex to larvae of *Tipula oleracea* (Diptera) and *Galleria mellonella* (Lepidoptera). J. Invertebr. Pathol. 69, 212–217. doi: 10.1006/jipa.1996.4647, PMID: 9170346

[B40] Eleftherianos I. Boundy S. Joyce S. A. Aslam S. Marshall J. W. Cox R. J. . (2007). An antibiotic produced by an insect-pathogenic bacterium suppresses host defenses through phenoloxidase inhibition. Proc. Natl. Acad. Sci. 104, 2419–2424. doi: 10.1073/pnas.0610525104, PMID: 17284598 PMC1892976

[B41] Fallet P. Bazagwira D. Ruzzante L. Ingabire G. Levivier S. Bustos-Segura C. . (2024). Entomopathogenic nematodes as an effective and sustainable alternative to control the fall armyworm in Africa. PNAS. Nexus. 3, 122. doi: 10.1093/pnasnexus/pgae122, PMID: 38628598 PMC11020222

[B42] Fang X. Zhang M. Tang Q. Wang Y. Zhang X. (2014). Inhibitory effect of *Xenorhabdus nematophila* TB on plant pathogens *Phytophthora capsici* and *Botrytis cinerea in vitro* and in planta. Sci. Rep. 4, 4300. doi: 10.1038/srep04300, PMID: 24599183 PMC3944712

[B43] Felföldi G. Marokházi J. Képiró M. Venekei I. (2009). Identification of natural target proteins indicates functions of a serralysin-type metalloprotease, PrtA, in anti-immune mechanisms. Appl. Environ. Microbiol. 75, 3120–3126. doi: 10.1128/AEM.02271-08, PMID: 19304826 PMC2681659

[B44] ffrench-Constant R. H. Dowling A. Waterfield N. R. (2007). Insecticidal toxins from *Photorhabdus* bacteria and their potential use in agriculture. Toxicon 49, 436–451. doi: 10.1016/j.toxicon.2006.11.019, PMID: 17207509

[B45] Filgueiras C. C. Willett D. S. Junior A. M. Pareja M. Borai F. E. Dickson D. W. . (2016). Stimulation of the salicylic acid pathway aboveground recruits entomopathogenic nematodes belowground. PloS One 11, e0154712. doi: 10.1371/journal.pone.0154712, PMID: 27136916 PMC4854467

[B46] Forst S. Dowds B. Boemare N. Stackebrandt E. (1997). *Xenorhabdus* and *Photorhabdus* spp.: Bugs that kill bugs. Annu. Rev. Microbiol. 51, 47–72. doi: 10.1146/annurev.micro.51.1.47, PMID: 9343343

[B47] Furgani G. Böszörményi E. Fodor A. Máthé-Fodor A. Forst S. Hogan J. S. . (2008). *Xenorhabdus* antibiotics: A comparative analysis and potential utility for controlling mastitis caused by bacteria. J. Appl. Microbiol. 104, 745–758. doi: 10.1111/j.1365-2672.2007.03613.x, PMID: 17976177

[B48] Gal T. Z. Glazer I. Koltai H. (2003). Differential gene expression during desiccation stress in the insect-killing nematode *Steinernema feltiae* IS-6. J. Parasitol. 89, 761–766. doi: 10.1645/GE-3105, PMID: 14533688

[B49] Gaugler R. Campbell J. F. McGuire T. R. (1990). Fitness of a genetically improved entomopathogenic nematode. J. Invertebr. Pathol. 56, 106–116. doi: 10.1016/0022-2011(90)90151-U

[B50] Georgis R. (1990). “ Formulation and application technology,” in Entomopathogenic Nematodes in Biological Control, Eds. Gaugler R. Kaya H. K. ( CRC Press, Boca Raton, FL), 173–194.

[B51] Glazer I. Simões N. Eleftherianos I. Ramakrishnan J. Ment D. Toubarro D. . (2025). Entomopathogenic nematodes: Survival, virulence and immunity. J. Invertebr. Pathol. 212, 108363. doi: 10.1016/j.jip.2025.108363, PMID: 40412605

[B52] Godina G. Vandenbossche B. Schmidt M. Sender A. Tambe A. H. Touceda-González M. . (2023). Entomopathogenic nematodes for biological control of *Psylliodes chrysocephala* (Coleoptera: Chrysomelidae) in oilseed rape. J. Invertebr. Pathol. 197, 107894. doi: 10.1016/j.jip.2023.107894, PMID: 36754114

[B53] Grewal P. S. (1998). Formulations of entomopathogenic nematodes for storage and application. Nematol Res. Jpn. J. Nematol 28, 68–74. doi: 10.3725/jjn1993.28.supplement_68

[B54] Grewal P. S. (2000). Enhanced ambient storage stability of an entomopathogenic nematode through anhydrobiosis. Pest Manag. Sci. 56, 401–406. doi: 10.1002/(SICI)1526-4998(200005)56:5<401::AID-PS137>3.0.CO;2-4

[B55] Grewal P. (2002). “ Formulation and application technology,” in Entomopathogenic Nematology. Ed. Gaugler R. ( CABI, Oxfordshire), 265–287.

[B56] Grewal P. S. Bornstein-Forst S. Burnell A. M. Glazer I. Jagdale G. B. (2006). Physiological, genetic, and molecular mechanisms of chemoreception, thermobiosis, and anhydrobiosis in entomopathogenic nematodes. Biol. Control. 38, 54–65. doi: 10.1016/j.biocontrol.2005.09.004

[B57] Grewal P. S. Jagdale G. B. (2002). Enhanced trehalose accumulation and desiccation survival of entomopathogenic nematodes through cold preacclimation. Biocontrol Sci. Technol. 12, 533–545. doi: 10.1080/0958315021000016207

[B58] Grunseich J. M. Aguirre N. M. Thompson M. N. Ali J. G. Helms A. M. (2021). Chemical cues from entomopathogenic nematodes vary across three species with different foraging strategies, triggering different behavioral responses in prey and competitors. J. Chem. Ecol. 47, 822–833. doi: 10.1007/s10886-021-01304-8, PMID: 34415500 PMC8613145

[B59] Guillermin M. L. Carrillo M. A. Hallem E. A. (2017). A single set of interneurons drives opposite behaviors in *C. elegans*. Curr. Biol. CB. 27, 2630–2639.e6. doi: 10.1016/j.cub.2017.07.023, PMID: 28823678 PMC6193758

[B60] Gulcu B. Hazir S. Kaya H. K. (2012). Scavenger deterrent factor (SDF) from symbiotic bacteria of entomopathogenic nematodes. J. Invertebr. Pathol. 110, 326–333. doi: 10.1016/j.jip.2012.03.014, PMID: 22446508

[B61] Gulcu B. Hazir S. Lewis E. E. Kaya H. K. (2018). Evaluation of responses of different ant species (Formicidae) to the scavenger deterrent factor associated with the entomopathogenic nematode-bacterium complex. Eur. J. Entomol. 115, 312–317. doi: 10.14411/eje.2018.030

[B62] Gumus A. Karagoz M. Shapiro-Ilan D. Hazir S. (2015). A novel approach to biocontrol: Release of live insect hosts pre-infected with entomopathogenic nematodes. J. Invertebr. Pathol. 130, 56–60. doi: 10.1016/j.jip.2015.07.002, PMID: 26149819

[B63] Hallem E. A. Dillman A. R. Hong A. V. Zhang Y. Yano J. M. DeMarco S. F. . (2011). A sensory code for host seeking in parasitic nematodes. Curr. Biol. 21, 377–383. doi: 10.1016/j.cub.2011.01.048, PMID: 21353558 PMC3152378

[B64] Han R. Ehlers R. U. (2000). Pathogenicity, development, and reproduction of *Heterorhabditis bacteriophora* and *Steinernema carpocapsae* under axenic *in vivo* conditions. J. Invertebr. Pathol. 75, 55–58. doi: 10.1006/jipa.1999.4900, PMID: 10631058

[B65] Hart A. C. Chao M. Y. (2010). " From odors to behaviors in Caenorhabditis elegans." in The Neurobiology of Olfaction. Ed. A. Menini (CRC Press, Boca Raton, FL), 1–35. 21882435

[B66] Hashmi S. Hashmi G. Glazer I. Gaugler R. (1998). Thermal response of *Heterorhabditis bacteriophora* transformed with the *Caenorhabditis elegans* hsp70 encoding gene. J. Exp. Zool. 281, 164–170. doi: 10.1002/(SICI)1097-010X(19980615)281:3<164::AID-JEZ2>3.0.CO;2-L, PMID: 9621437

[B67] Hazir S. Shapiro-Ilan D. I. Bock C. H. Hazir C. Leite L. G. Hotchkiss M. W. (2016). Relative potency of culture supernatants of *Xenorhabdus* and *Photorhabdus* spp. on growth of some fungal phytopathogens. Eur. J. Plant Pathol. 146, 369–381. doi: 10.1007/s10658-016-0923-9

[B68] Helms A. M. Ray S. Matulis N. L. Kuzemchak M. C. Grisales W. Tooker J. F. . (2019). Chemical cues linked to risk: Cues from below-ground natural enemies enhance plant defences and influence herbivore behaviour and performance. Funct. Ecol. 33, 798–808. doi: 10.1111/1365-2435.13297

[B69] Hiltpold I. Hibbard B. E. French B. W. Turlings T. C. J. (2012). Capsules containing entomopathogenic nematodes as a Trojan horse approach to control the western corn rootworm. Plant Soil. 358, 11–25. doi: 10.1007/s11104-012-1253-0

[B70] Hinchliffe S. J. Hares M. C. Dowling A. J. Ffrench-Constant R. H. (2010). Insecticidal toxins from the Photorhabdus and *Xenorhabdus* bacteria. Open Toxinol. J. 3, 83–100. doi: 10.2174/1875414701003010101

[B71] Hussein M. Abdel-Aty M. (2012). Formulation of two native entomopathogenic nematodes at room temperature. J. Biopestic. 5, 23–27. doi: 10.57182/jbiopestic.5.0.23-27

[B72] Hwang J. Park Y. Kim Y. Hwang J. Lee D. (2013). An entomopathogenic bacterium, *Xenorhabdus nematophila*, suppresses expression of antimicrobial peptides controlled by Toll and Imd pathways by blocking eicosanoid biosynthesis. Arch. Insect Biochem. Physiol. 83, 151–169. doi: 10.1002/arch.21103, PMID: 23740621

[B73] Jagdale G. B. Grewal P. S. (2003). Acclimation of entomopathogenic nematodes to novel temperatures: Trehalose accumulation and the acquisition of thermotolerance. Int. J. Parasitol. 33, 145–152. doi: 10.1016/S0020-7519(02)00257-6, PMID: 12633652

[B74] Jagdale G. B. Kamoun S. Grewal P. S. (2009). Entomopathogenic nematodes induce components of systemic resistance in plants: Biochemical and molecular evidence. Biol. Control. 51, 102–109. doi: 10.1016/j.biocontrol.2009.06.009

[B75] Kajuga J. N. Waweru B. W. Bazagwira D. Ishimwe P. M. Ndacyayisaba S. Mukundiyabo G. C. . (2025). Efficacy of foliar applications of entomopathogenic nematodes in the management of the invasive tomato leaf miner *Phthorimaea absoluta* compared to local practices under open-field conditions. Agronomy 15, 1417. doi: 10.3390/agronomy15061417

[B76] Kallali N. S. Ouijja A. Goura K. Laasli S. E. Kenfaoui J. Benseddik Y. . (2024). From soil to host: Discovering the tripartite interactions between entomopathogenic nematodes, symbiotic bacteria and insect pests and related challenges. J. Nat. Pestic. Res. 7, 100065. doi: 10.1016/j.napere.2023.100065

[B77] Karimi S. Eivazian Kary N. Mohammadi D. (2025). Optimization of carboxymethyl cellulose-based formulations for enhanced shelf life of an entomopathogenic nematode, Steinernema carpocapsae. Egypt. J. Biol. Pest Control. 35, 23. doi: 10.1186/s41938-025-00858-z

[B78] Kary N. E. Chahardoli S. Mohammadi D. Dillon A. B. (2021). Efficacy of carboxymethyl cellulose as an inert water-soluble carrier for formulation of entomopathogenic nematodes, *Heterorhabditis bacteriophora* and Steinernema carpocapsae. Biol. Control. 160, 104690. doi: 10.1016/j.biocontrol.2021.104690

[B79] Kim J. Hiltpold I. Jaffuel G. Sbaiti I. Hibbard B. E. Turlings T. C. J. (2021). Calcium-alginate beads as a formulation for the application of entomopathogenic nematodes to control rootworms. J. Pest Sci. 94, 1197–1208. doi: 10.1007/s10340-021-01349-4, PMID: 34720786 PMC8550308

[B80] Kim J. Jaffuel G. Turlings T. C. J. (2015). Enhanced alginate capsule properties as a formulation of entomopathogenic nematodes. BioControl 60, 527–535. doi: 10.1007/s10526-014-9638-z

[B81] Koppenhöfer A. M. Luiza Sousa A. (2024). Long-term suppression of turfgrass insect pests with native persistent entomopathogenic nematodes. J. Invertebr. Pathol. 204, 108123. doi: 10.1016/j.jip.2024.108123, PMID: 38705354

[B82] Koppenhöfer A. M. Shapiro-Ilan D. I. Hiltpold I. (2020). Entomopathogenic nematodes in sustainable food production. Front. Sustain. Food Syst. 4. doi: 10.3389/fsufs.2020.00125

[B83] Kotliarevski L. Cohen R. Ramakrishnan J. Wu S. Mani K. A. Amar-Feldbaum R. . (2022). Individual coating of entomopathogenic nematodes with titania (TiO2) nanoparticles based on oil-in-water pickering emulsion: a new formulation for biopesticides. J. Agric. Food Chem. 70, 13518–13527. doi: 10.1021/acs.jafc.2c04424, PMID: 36226658

[B84] Krishnayyaand P. V. Grewal P. S. (2002). Effect of neem and selected fungicides on viability and virulence of the entomopathogenic nematode Steinernema feltiae. Biocontrol Sci. Technol. 12, 259–266. doi: 10.1080/09583150210388

[B85] Lacey L. A. Georgis R. (2012). Entomopathogenic nematodes for control of insect pests above and below ground with comments on commercial production. J. Nematol. 44, 218–225., PMID: 23482993 PMC3578470

[B86] Laznik Ž. Košir I. J. Košmelj K. Murovec J. Jagodič A. Trdan S. . (2020). Effect of *Cannabis sativa* L. root, leaf and inflorescence ethanol extracts on the chemotrophic response of entomopathogenic nematodes. Plant Soil. 455, 367–379. doi: 10.1007/s11104-020-04693-z

[B87] Laznik Ž. Trdan S. (2016). Attraction behaviors of entomopathogenic nematodes (Steinernematidae and Heterorhabditidae) to synthetic volatiles emitted by insect damaged potato tubers. J. Chem. Ecol. 42, 314–322. doi: 10.1007/s10886-016-0686-y, PMID: 27108451

[B88] Leite L. G. Shapiro-Ilan D. I. Hazir S. (2018). Survival of *Steinernema feltiae* in different formulation substrates: Improved longevity in a mixture of gel and vermiculite. Biol. Control. 126, 192–197. doi: 10.1016/j.biocontrol.2018.05.013

[B89] Liao C. Zhang S. Fu T. Jin X. Xu W. Liu D. . (2025). Mutualistic bacteria of entomopathogenic nematodes as an insecticidal agent for sustainable agriculture. Chem. Biol. Technol. Agric. 12, 141. doi: 10.1186/s40538-025-00862-3

[B90] Liu J. Li W. Wang C. Liang H. Chu Z. Wang G. . (2025). Mixtures of entomopathogenic nematodes provided successful suppression of *Bactrocera tau* by stimulated dispersal, aggregation, and invasion. Biol. Control. 208, 105858. doi: 10.1016/j.biocontrol.2025.105858

[B91] Llácer E. Martínez de Altube M. M. Jacas J. A. (2009). Evaluation of the efficacy of *Steinernema carpocapsae* in a chitosan formulation against the red palm weevil, *Rhynchophorus ferrugineus*, in Phoenix canariensis. BioControl 54, 559–565. doi: 10.1007/s10526-008-9208-3 19924729

[B92] Lortkipanidze M. A. Gorgadze O. A. Kajaia G. Gratiashvili N. G. Kuchava M. A. (2016). Foraging behavior and virulence of some entomopathogenic nematodes. Ann. Agrar. Sci. 2, 99–103. doi: 10.1016/j.aasci.2016.05.009

[B93] Lu D. Macchietto M. Chang D. Barros M. M. Baldwin J. Mortazavi A. . (2017). Activated entomopathogenic nematode infective juveniles release lethal venom proteins. PloS Pathog. 13, e1006302. doi: 10.1007/s00248-020-01573-y, PMID: 28426766 PMC5398726

[B94] Maher A. M. D. Asaiyah M. Quinn S. Burke R. Wolff H. Bode H. B. . (2021). Competition and co-existence of two *Photorhabdus* symbionts with a nematode host. Microb. Ecol. 81, 223–239., PMID: 32827089 10.1007/s00248-020-01573-y

[B95] Mahmoud M. F. Mahfouz H. M. Mohamed K. M. (2016). Compatibility of entomopathogenic nematodes with neonicotinoids and Azadirachtin insecticides for controlling the black cutworm, *Agrotis ipsilon* (Hufnagel) in canola plants. IJRES 2, 11–18. doi: 10.20431/2454-9444.0201002

[B96] Manohar M. Tenjo-Castano F. Chen S. Zhang Y. K. Kumari A. Williamson V. M. . (2020). Plant metabolism of nematode pheromones mediates plant-nematode interactions. Nat. Commun. 11, 208. doi: 10.1038/s41467-019-14104-2, PMID: 31924834 PMC6954178

[B97] Matadamas-Ortiz P. T. Ruiz-Vega J. Vazquez-Feijoo J. A. Cruz-Martínez H. Cortés-Martínez C. I. (2014). Mechanical production of pellets for the application of entomopathogenic nematodes: Factors that determine survival time of Steinernema glaseri. Biocontrol Sci. Technol. 24, 145–157. doi: 10.1080/09583157.2013.852161

[B98] McMullen J. G. Peterson B. F. Forst S. Blair H. G. Stock S. P. (2017). Fitness costs of symbiont switching using entomopathogenic nematodes as a model. BMC Evol. Biol. 17, 100. doi: 10.1186/s12862-017-0939-6, PMID: 28412935 PMC5392933

[B99] Metwally H. M. S. Saleh M. M. E. Abonaem M. (2025). Formulation for foliar and soil application of entomopathogenic nematodes for controlling the onion thrips *Thrips tabaci* Lindeman (Thysanoptera: Thripidae). Egypt. J. Biol. Pest Control. 35, 4. doi: 10.1186/s41938-025-00841-8

[B100] Modic Š. Žigon P. Kolmanič A. Trdan S. Razinger J. (2020). Evaluation of the field efficacy of *Heterorhabditis bacteriophora* poinar (Rhabditida: Heterorhabditidae) and synthetic insecticides for the control of western corn rootworm larvae. Insects 11. doi: 10.3390/insects11030202, PMID: 32213940 PMC7143195

[B101] Moore S. D. Ehlers R. U. Manrakhan A. Gilbert M. Kirkman W. Daneel J. H. . (2024). Field-scale efficacy of entomopathogenic nematodes to control false codling moth, *Thaumatotibia leucotreta* (Lepidoptera: Tortricidae), in citrus orchards in South Africa. Crop Prot. 179, 106610. doi: 10.1016/j.cropro.2024.106610

[B102] Moreira G. F. Batista E. S. D. P. Campos H. B. N. Lemos R. E. Ferreira M. D. C. (2013). Spray nozzles, pressures, additives and stirring time on viability and pathogenicity of entomopathogenic nematodes (Nematoda: Rhabditida) for greenhouses. PloS One 8, e65759. doi: 10.1371/journal.pone.0065759, PMID: 23755280 PMC3675033

[B103] Navon A. Keren S. Salame L. Glazer I. (1998). An edible-to-insects calcium alginate gel as a carrier for entomopathogenic nematodes. Biocontrol Sci. Technol. 8, 429–437. doi: 10.1080/09583159830225

[B104] Navon A. Nagalakshmi V. K. Levski S. Salame L. Glazer I. (2002). Effectiveness of entomopathogenic nematodes in an alginate gel formulation against Lepidopterous pests. Biocontrol Sci. Technol. 12, 737–746. doi: 10.1080/0958315021000039914

[B105] O’Halloran D. M. Burnell A. M. (2003). An investigation of chemotaxis in the insect parasitic nematode *Heterorhabditis bacteriophora*. Parasitology 127, 375–385. doi: 10.1017/S0031182003003688, PMID: 14636024

[B106] Odendaal D. Addison M. F. Malan A. P. (2016). Entomopathogenic nematodes for the control of the codling moth (*Cydia pomonella* L.) in field and laboratory trials. J. Helminthol. 90, 615–623. doi: 10.1017/S0022149X15000887, PMID: 26484481

[B107] Ozawa K. Shinkai Y. Kako K. Fukamizu A. Doi M. (2022). The molecular and neural regulation of ultraviolet light phototaxis and its food-associated learning behavioral plasticity in C. elegans. Neurosci. Lett. 770, 136384. doi: 10.1016/j.neulet.2021.136384, PMID: 34890717

[B108] Park Y. Kim Y. (2000). Eicosanoids rescue *Spodoptera exigua* infected with *Xenorhabdus nematophilus*, the symbiotic bacteria to the entomopathogenic nematode Steinernema carpocapsae. J. Insect Physiol. 46, 1469–1476. doi: 10.1016/S0022-1910(00)00071-8, PMID: 10891575

[B109] Perez E. E. Lewis E. E. Shapiro-Ilan D. I. (2003). Impact of the host cadaver on survival and infectivity of entomopathogenic nematodes (Rhabditida: Steinernematidae and Heterorhabditidae) under desiccating conditions. J. Invertebr. Pathol. 82, 111–118. doi: 10.1016/S0022-2011(02)00204-5, PMID: 12623311

[B110] Perier J. D. Wu S. Arthurs S. P. Toews M. D. Shapiro-Ilan D. I. (2025). Persistence of the entomopathogenic nematode *Steinernema feltiae* in a novel capsule formulation. Biol. Control. 200, 105684. doi: 10.1016/j.biocontrol.2024.105684

[B111] Pervez R. Lone S. A. Pattnaik S. (2020). Characterization of symbiotic and associated bacteria from entomopathogenic nematode Heterorhabditis sp. (nematode: Heterorhabditidae) isolated from India. Egypt. J. Biol. Pest Control. 30. doi: 10.1186/s41938-020-00343-9

[B112] Platt T. Stokwe N. F. Malan A. P. (2018). Foliar application of *Steinernema yirgalemense* to control *Planococcus ficus*: Assessing adjuvants to improve efficacy. South Afr. J. Enol. Vitic. 40. doi: 10.21548/40-1-2920

[B113] Polat B. Cengiz A. Koc S. Kokten S. K. Gultekin Z. N. Caliskan C. . (2024). Efficacy of *Steinernema feltiae* nematode against lesser mealworm (*Alphitobius diaperinus*) populations from poultry farms in Türkiye. Vet. Sci. 11, 567. doi: 10.3390/vetsci11110567, PMID: 39591341 PMC11598855

[B114] Půža V. Tarasco E. (2023). Interactions between entomopathogenic fungi and entomopathogenic nematodes. Microorganisms 11, 163. doi: 10.3390/microorganisms11010163, PMID: 36677455 PMC9864569

[B115] Raja R. Hazir C. Gümüş A. Asan C. Karagöz M. Hazir S. (2015). Efficacy of the entomopathogenic nematode *Heterorhabditis bacteriophora* using different application methods in the presence or absence of a natural enemy. Turk. J. Agric. For. 39, 277–285. doi: 10.3906/tar-1410-33

[B116] Ramakrishnan J. Salame L. Mani K. A. Feldbaum R. Karavani E. Mechrez G. . (2023). Increasing the survival and efficacy of entomopathogenic nematodes on exposed surfaces by Pickering emulsion formulations offers new venue for foliar pest management. J. Invertebr. Pathol. 199, 107938. doi: 10.1016/j.jip.2023.107938, PMID: 37268287

[B117] Ramakuwela T. Hatting J. Laing M. D. Hazir S. Thiebaut N. (2016). *In vitro* solid-state production of *Steinernema innovationi* with cost analysis. Biocontrol Sci. Technol. 26, 792–808. doi: 10.1080/09583157.2016.1159284

[B118] Ramakuwela T. Tarasco E. Chavarría-Hernández N. Toepfer S. (2025). Entomopathogenic nematodes: Commercial use and future perspectives. J. Invertebr. Pathol. 212, 108388. doi: 10.1016/j.jip.2025.108388, PMID: 40532927

[B119] Rasmann S. Köllner T. G. Degenhardt J. Hiltpold I. Toepfer S. Kuhlmann U. . (2005). Recruitment of entomopathogenic nematodes by insect-damaged maize roots. Nature 434, 732–737. doi: 10.1038/nature03451, PMID: 15815622

[B120] Sabino PH de S. Sales F. S. Guevara E. J. Junior A. M. Filgueiras C. C. (2014). Compatibility of entomopathogenic nematodes (Nematoda: Rhabditida) with insecticides used in the tomato crop. Nematoda 1, e03014. doi: 10.4322/nematoda.03014

[B121] Sáenz-Aponte A. Correa-Cuadros J. P. Rodríguez-Bocanegra M. X. (2020). Foliar application of entomopathogenic nematodes and fungi for the management of the diamondback moth in greenhouse and field. Biol. Control. 142, 104163. doi: 10.1016/j.biocontrol.2019.104163

[B122] San-Blas E. (2013). Progress on entomopathogenic nematology research: A bibliometric study of the last three decades: 1980–2010. Biol. Control. 66, 102–124. doi: 10.1016/j.biocontrol.2013.04.002

[B123] Schroer S. Yi X. Ehlers R. U. (2005). Evaluation of adjuvants for foliar application of *Steinernema carpocapsae* against larvae of the diamondback moth (*Plutella xylostella*). Nematology 7, 37–44. doi: 10.1163/1568541054192126

[B124] Shapiro D. I. Behle R. McGuire M. R. Lewis E. E. (2003). Formulated arthropod cadavers for pest suppression. U.S. Patent No. 6,524,601. Washington, DC: U.S. Patent and Trademark Office.

[B125] Shapiro D. I. Glazer I. (1996). Comparison of entomopathogenic nematode dispersal from infected hosts versus aqueous suspension. Environ. Entomol. 25, 1455–1461. doi: 10.1093/ee/25.6.1455

[B126] Shapiro D. I. Glazer I. Segal D. (1997). Genetic improvement of heat tolerance in *Heterorhabditis bacteriophora* through hybridization. Biol. Control. 8, 153–159. doi: 10.1006/bcon.1996.0488

[B127] Shapiro D. I. Lewis E. E. (1999). Comparison of entomopathogenic nematode infectivity from infected hosts versus aqueous suspension. Environ. Entomol. 28, 907–911. doi: 10.1093/ee/28.5.907

[B128] Shapiro-Ilan D. I. Campbell J. F. Lewis E. E. Elkon J. M. Kim-Shapiro D. B. (2009). Directional movement of Steinernematid nematodes in response to electrical current. J. Invertebr. Pathol. 100, 134–137. doi: 10.1016/j.jip.2008.11.001, PMID: 19041325

[B129] Shapiro-Ilan D. I. Cottrell T. E. Mizell R. F. Horton D. L. Behle R. W. Dunlap C. A. (2010a). Efficacy of *Steinernema carpocapsae* for control of the lesser peachtree borer, *Synanthedon pictipes*: Improved aboveground suppression with a novel gel application. Biol. Control. 54, 23–28. doi: 10.1016/j.biocontrol.2009.11.009

[B130] Shapiro-Ilan D. Dolinski C. (2015). Entomopathogenic nematode application technology. Nematode. Pathog. Insects. Pests. Ecol. Appl. Technol. Sustain. Plant Crop Prot. 231–254. doi: 10.1007/978-3-319-18266-7_9

[B131] Shapiro-Ilan D. I. Goolsby J. A. (2021). Evaluation of Barricade^®^ to enhance survival of entomopathogenic nematodes on cowhide. J. Invertebr. Pathol. 184, 107592. doi: 10.1016/j.jip.2021.107592, PMID: 33882276

[B132] Shapiro-Ilan D. I. Lewis E. E. Behle R. W. McGuire M. R. (2001). Formulation of entomopathogenic nematode-infected cadavers. J. Invertebr. Pathol. 78, 17–23. doi: 10.1006/jipa.2001.5030, PMID: 11500089

[B133] Shapiro-Ilan D. I. Lewis E. E. Campbell J. F. Kim-Shapiro D. B. (2012). Directional movement of entomopathogenic nematodes in response to electrical field: Effects of species, magnitude of voltage, and infective juvenile age. J. Invertebr. Pathol. 109, 34–40. doi: 10.1016/j.jip.2011.09.004, PMID: 21945052

[B134] Shapiro-Ilan D. I. Lewis E. E. Son Y. Tedders W. L. (2003). Superior efficacy observed in entomopathogenic nematodes applied in infected-host cadavers compared with application in aqueous suspension. J. Invertebr. Pathol. Washington, DC: U.S. Patent and Trademark Office 83, 270–272. doi: 10.1016/S0022-2011(03)00101-0, PMID: 12877838

[B135] Shapiro-Ilan D. I. Morales-Ramos J. A. Rojas M. G. Tedders W. L. (2010b). Effects of a novel entomopathogenic nematode-infected host formulation on cadaver integrity, nematode yield, and suppression of *Diaprepes abbreviatus* and Aethina tumida. J. Invertebr. Pathol. 103, 103–108. doi: 10.1016/j.jip.2009.11.006, PMID: 19932701

[B136] Shields E. J. Testa A. M. O’Neil W. J. (2018). Long-term persistence of native New York entomopathogenic nematode isolates across crop rotation. J. Econ. Entomol. 111, 2592–2598. doi: 10.1093/jee/toy258, PMID: 30169810

[B137] Silver S. C. Dunlop D. B. Grove D. I. (1995). Granular formulation of entities with improved storage stability. WIPO Patent No. WO 95/0577. Geneva, CH: World Intellectual Property Organization.

[B138] Solomon A. Paperna I. Glazer I. (1999). Desiccation survival of the entomopathogenic nematode *Steinernema feltiae*: Induction of anhydrobiosis. Nematology 1, 61–68. doi: 10.1163/156854199507983

[B139] Solomon A. Salomon R. Paperna I. Glazer I. (2000). Desiccation stress of entomopathogenic nematodes induces the accumulation of a novel heat-stable protein. Parasitology 121, 409–416. doi: 10.1017/S0031182099006563, PMID: 11072904

[B140] Somvanshi V. S. Koltai H. Glazer I. (2008). Expression of different desiccation-tolerance related genes in various species of entomopathogenic nematodes. Mol. Biochem. Parasitol. 158, 65–71. doi: 10.1016/j.molbiopara.2007.11.012, PMID: 18179831

[B141] Srivastava C. N. Mohan L. Sharma P. Maurya P. (2011). A review on prospectives of synergistic approach in insect pest management. J. Entomol. Res. 35, 255–266.

[B142] Stilwell M. D. Cao M. Goodrich-Blair H. Weibel D. B. (2018). Studying the symbiotic bacterium *Xenorhabdus nematophila* in individual, living *Steinernema carpocapsae* nematodes using microfluidic systems. mSphere 3. doi: 10.1128/msphere.00530-17, PMID: 29299529 PMC5750387

[B143] Stock S. P. Campos-Herrera R. Shapiro-Ilan D. (2025). The first 100 years in the history of entomopathogenic nematodes. J. Invertebr. Pathol. 211, 108302. doi: 10.1016/j.jip.2025.108302, PMID: 40081791

[B144] Stock S. P. Kusakabe A. Orozco R. A. (2017). Secondary metabolites produced by *Heterorhabditis* symbionts and their application in agriculture: what we know and what to do next. J. Nematol. 49, 373. doi: 10.21307/jofnem-2017-084, PMID: 29353924 PMC5770283

[B145] Strauch O. Niemann I. Neumann A. Schmidt A. J. Peters A. Ehlers R. U. (2000). Storage and formulation of the entomopathogenic nematodes *Heterorhabditis indica* and H. bacteriophora. BioControl 45, 483–500. doi: 10.1023/A:1026528727365

[B146] Sugiyama T. Hasegawa K. (2025). Synergistic actions of symbiotic bacteria modulate the insecticidal potency of entomopathogenic nematode *Steinernema monticolum* KHA701. Sci. Rep. 15. doi: 10.1038/s41598-025-06488-7, PMID: 40594438 PMC12219376

[B147] Sumaya N. H. Gohil R. Okolo C. Addis T. Doerfler V. Ehlers R. U. . (2018). Applying inbreeding, hybridization and mutagenesis to improve oxidative stress tolerance and longevity of the entomopathogenic nematode Heterorhabditis bacteriophora. J. Invertebr. Pathol. 151, 50–58. doi: 10.1016/j.jip.2017.11.001, PMID: 29108857

[B148] Sun B. Zhang X. Song L. Zheng L. Wei X. Gu X. . (2021). Evaluation of indigenous entomopathogenic nematodes in Southwest China as potential biocontrol agents against *Spodoptera litura* (Lepidoptera: Noctuidae). J. Nematol. 53, e2021–e2083. doi: 10.21307/jofnem-2021-083, PMID: 34820628 PMC8609611

[B149] Tarasco E. Fanelli E. Salvemini C. El-Khoury Y. Troccoli A. Vovlas A. . (2023). Entomopathogenic nematodes and their symbiotic bacteria: From genes to field uses. Front. Insect Sci. 3, 1195254. doi: 10.3389/finsc.2023.1195254, PMID: 38469514 PMC10926393

[B150] Thakur N. Tomar P. Kaur S. Kumari P. (2022). Virulence of native entomopathogenic nematodes against major lepidopteran insect species of tomato (*Solanum lycopersicum* L.). J. Appl. Biol. Biotechnol. 10, 6–14. doi: 10.7324/JABB.2022.10s102

[B151] Tobias N. J. Wolff H. Djahanschiri B. Grundmann F. Kronenwerth M. Shi Y. M. . (2017). Natural product diversity associated with the nematode symbionts *Photorhabdus* and Xenorhabdus. Nat. Microbiol. 12, 1676–1685. doi: 10.1038/s41564-017-0039-9, PMID: 28993611

[B152] Tomar P. Thakur N. (2022). Biocidal potential of indigenous isolates of entomopathogenic nematodes (EPNs) against tobacco cutworm, *Spodoptera litura* Fabricius (Lepidoptera: Noctuidae). Egypt. J. Biol. Pest Control. 32, 107. doi: 10.1186/s41938-022-00607-6

[B153] Torr P. Heritage S. Wilson M. J. (2004). Vibrations as a novel signal for host location by parasitic nematodes. Int. J. Parasitol. 34, 997–999. doi: 10.1016/j.ijpara.2004.05.003, PMID: 15313127

[B154] Tumialis D. Mazurkiewicz A. Florczak L. Skrzecz I. (2023). The potential of entomopathogenic nematodes of the genera *Steinernema* and *Heterorhabditis* for biological control of the pine lappet moth *Dendrolimus pini* L. (Lepidoptera: Lasiocampidae) in Scots pine stands. For. Int. J. For. Res. 96, 733–739. doi: 10.1093/forestry/cpad008

[B155] United Nations (2017). World population prospects: The 2017 revision (New York, USA: United Nations Department of Economic and Social Affairs), 24.

[B156] Valle E. E. D. Dolinski C. Barreto E. L. S. Souza R. M. (2009). Effect of cadaver coatings on emergence and infectivity of the entomopathogenic nematode *Heterorhabditis baujardi* LPP7 (Rhabditida: Heterorhabditidae) and the removal of cadavers by ants. Biol. Control. 50, 21–24. doi: 10.1016/j.biocontrol.2009.01.007

[B157] Vellai T. Molnár A. Lakatos L. Bánfalvi Z. Fodor A. Sáringer G. (1999). Transgenic nematodes carrying a cloned stress resistance gene from yeast. Surviv. Entomopathog. Nematodes. Off. Off. Publ. Eur. Commun. Luxemb., 105–119.

[B158] Wang Y. Gaugler R. (1999). *Steinernema glaseri* surface coat protein suppresses the immune response of *Popillia japonica* (Coleoptera: Scarabaeidae) larvae. Biol. Control. 14, 45–50. doi: 10.1006/bcon.1998.0672

[B159] Wang A. Tang H. Sun J. Wang L. Rasmann S. Ruan W. . (2025). Entomopathogenic nematodes-killed insect cadavers in the rhizosphere activate plant direct and indirect defences aboveground. Plant Cell Environ. 48, 923–939. doi: 10.1111/pce.15193, PMID: 39370758

[B160] Wu H. Gong Q. Fan K. Sun R. Xu Y. Zhang K. (2017). Synergistic effect of entomopathogenic nematodes and thiamethoxam in controlling *Bradysia odoriphaga* Yang and Zhang (Diptera: Sciaridae). Biol. Control. 111, 53–60. doi: 10.1016/j.biocontrol.2017.05.006

[B161] Yadav A. K. Lalramliana (2012). Efficacy of indigenous entomopathogenic nematodes from Meghalaya, India against the larvae of taro leaf beetle, *Aplosonyx chalybaeus* (Hope). J. Parasit. Dis. 36, 149–154. doi: 10.1007/s12639-012-0139-7, PMID: 24082518 PMC3427670

[B162] Yukawa T. Pitt J. M. (1985). Nematode storage and transport. WIPO Patent No. WO 85/03412. Geneva, CH: World Intellectual Property Organization.

[B163] Zhang X. Li L. Kesner L. Robert C. A. M. (2021). Chemical host-seeking cues of entomopathogenic nematodes. Curr. Opin. Insect Sci. 44, 72–81. doi: 10.1016/j.cois.2021.03.011, PMID: 33866041

[B164] Zulu S. Ramakuwela T. Baimey H. Laing M. Shapiro-Ilan D. Cochrane N. (2025). Storage capacity of entomopathogenic nematodes in Barricade^®^ gel and potassium polyacrylate hydrogel. J. Nematol. 57. doi: 10.2478/jofnem-2025-0014, PMID: 40547568 PMC12182831

